# Elevated miRNA Inversely Correlates with *E-cadherin* Gene Expression in Tissue Biopsies from Crohn Disease Patients in contrast to Ulcerative Colitis Patients

**DOI:** 10.1155/2020/4250329

**Published:** 2020-07-22

**Authors:** Małgorzata Guz, Tomasz Dworzański, Witold Jeleniewicz, Marek Cybulski, Joanna Kozicka, Andrzej Stepulak, Krzysztof Celiński

**Affiliations:** ^1^Department of Biochemistry and Molecular Biology, Medical University of Lublin, 20-093 Lublin, Poland; ^2^Department of Gastroenterology with Endoscopic Unit, Medical University of Lublin, 20-090 Lublin, Poland

## Abstract

Inflammatory bowel disease (IBD) comprises ulcerative colitis (UC) and Crohn disease (CD). Similar symptoms, but different treatment procedures for both diseases require precise diagnosis. MicroRNAs (miRNAs) are major posttranscriptional players that regulate the expression of genes during the inflammation and thus could be appropriate biomarkers for differentiation between UC and CD. For this purpose, we analyzed the expression of *miR-21-3p*, *miR-31-3p*, *miR-125b-1-3p*, *miR-146a-3p*, *miR-155-5p*, and *E-cadherin* (*CDH1)* genes associated with IBD, in 67 tissue samples: 28 inflamed mucosa samples (*n* = 16 UC, *n* = 12 CD), 28 adjacent normal colonic mucosa (*n* = 16 UC, *n* = 12 CD), and 11 normal mucosa from healthy patients using reverse transcription real-time RT-PCR. We found all analyzed miRNAs were significantly overexpressed in UC tissue as compared to adjacent normal tissue of patients with UC, as well as to normal mucosa from healthy controls. Four miRNAs (except *miR-125b-1-3p*) were significantly upregulated in CD lesions as compared to adjacent normal tissue of patients with CD, and four miRNAs, except *miR-146a-3p*, were significantly higher in CD samples compared to normal mucosa from healthy individuals. In the CD group, we found an inverse correlation between *miR-155-5p* or *miR-146a-3p* expressions and *CDH1*expression in inflamed mucosa. This type of correlation was also detected for *miR-213p* in adjacent normal tissue and *CDH1* in inflamed mucosa, as well as between *miR-155-5p* and *CDH1* in adjacent normal tissue. Elevated miRNA expression is characteristic for IBD-mediated inflammation process and inversely correlated with *CDH1* gene expression, which suggest involvement of epithelial to mesenchymal transition (EMT) in IBD development.

## 1. Introduction

IBD is a group of chronic disorders that occur in the form of ulcerative colitis (UC) and Crohn disease (CD) [1]. Inflammatory changes in CD involve all the layers of the intestinal wall and can develop in any part of the digestive tract, predominantly in the final section of the small intestine and in the large intestine [2]. UC is characterized by continuous inflammatory changes within the mucous membrane and affects only the large intestine [3]. More than 2 million Europeans and 1.5 million North Americans are suffering with IBD [4]. The incidence of IBD is increasing in newly industrialized nations, which is associated with nutrition consumption of processed food, refined sugars, dairy, and lower intake of plant-based fibers [5]. However, the etiology of IBD still remains unknown. Genome-wide association studies (GWAS) support a role of heredity in the pathogenesis of IBD that accounts 23% and 16% in CD and UC, respectively [6]. In individuals with genetic risk, abnormal interactions between the host immune system and gut flora, as well as dysregulation of cellular responses such as autophagy and endoplasmic reticulum stress, induce an immune response in the gut resulting in intestinal inflammation [7]. Other possible reasons for IBD development include influence of other genetic, and especially epigenetic, changes including microRNA-mediated regulation of gene expression [8]. MicroRNAs (miRNAs) are an abundant class of endogenous, small, single-stranded, noncoding RNAs composed of 18-25 nucleotides formed from double stranded precursors. These small molecular players repress protein translation through binding to complementary 3′ untranslated region (UTR), 5′ UTR, or coding sequence of targeted mRNAs [9]. miRNAs play a major role in a wide range of developmental process including cell proliferation, cell cycle, cell differentiation, metabolism, apoptosis, developmental timing, neuronal cell fate, neuronal gene expression, brain morphogenesis, muscle differentiation, and stem cell division [10]. Many miRNAs display organ-specific expression patterns suggesting cell-type-specific functions [11]. Abnormal expression of miRNAs can lead to pathological processes involving the immune system such as carcinogenesis and autoimmunity [12]. In addition, miRNAs are stable, easily isolated from different types of tissues, as well as from blood, which allow to consider them as an ideal biological marker [13]. The aim of our study was to determine and compare the expression of five microRNAs (*miR-155-5p*, *miR-21-3p*, *miR-31-3p*, *miR-146a-3p*, and *miR-125b-1-3p*) in tissue specimens of patients with active CD and UC. These miRNAs were selected for analysis, because of their involvement in the regulation of gene expression responsible for the development and progression of IBD [14–16]. We evaluated the usefulness of these miRNAs as potential biomarkers for inflammatory process during IBD and possibly to differentiate between UC and CD. For that purpose, we applied miRWalk algorithm analysis to assess the possible miRNA-target interactions (http://mirwalk.umm.uni-heidelberg.de) with genes involved in IBD pathology. We determined the expression of E-cadherin (*CDH1*), a major transmembrane glycoprotein important for the maintenance for intestinal epithelial homeostasis [17]. E-cadherin is essential for proper morphogenesis of intestine, because it is a core component of adherent junctions, the major cell-cell adhesion structure, in which disruption may lead to inflammation and cancer [18]. Polymorphisms of *CDH1* result in abnormal protein localization and may be involved in pathogenesis of IBD which disturb intestinal homeostasis [19]. We aimed to correlate expression of *CDH1* gene with miRNA expression patterns in order to evaluate their potential role in IBD development. Since there is still lack of standard methods that help to distinguish between UC and CD, our present study could contribute to the improvement of the accuracy of the diagnosis together with different endoscopic techniques.

## 2. Materials and Methods

### 2.1. Patients

The selection of patients from the CD and UC groups follows the endoscopic evaluation of the severity of inflammatory lesions according to the Mayo endoscopic score for UC and the Simple Endoscopic Score for CD (SES-CD). The research was conducted in accordance with the Declaration of Helsinki (1964), and the local ethical committee approved the study design (No. KE-0254/2013). The biological material was collected during colonoscopies performed in the Endoscopic Unit of the Department of Gastroenterology, Medical University of Lublin, Poland.

The inclusion criteria of patients enrolled in our study are as follows: diagnosed and histopathologically confirmed UC and CD, age 18-65, and active inflammatory changes assessed >2 points on the Mayo scale for UC and >3 points on the SES-CD for CD. The exclusion criteria are as follows: gastrointestinal infections caused by *Clostridium difficile*, *Salmonella*, *Shigella*, *Campylobacter*, and *Yersinia*; ischemic changes, inflammation following X-ray therapy; and oral administration of antibiotics and probiotics.

The patients were divided into following groups: the group of 12 patients diagnosed with CD, and the group of 16 patients with UC, and two biopsy specimens: from the inflammation-altered tissue and the normal tissue collected from each patient. The control group consisted of 11 patients without any inflammatory lesions found on colonoscopies performed within the screening program. In this group, the biopsy specimens of the normal tissue were collected. Patients' characteristic is depicted in Table 1.

### 2.2. Sample Handling, Isolation of RNA, and Synthesis of cDNA

Immediately after the collection of tissue biopsies, the specimens were placed in RNAlater™ (Sigma Aldrich, St. Louis, MO) and stored at -80°C for further RNA isolation.

Total RNA including miRNAs were isolated using miRCURY RNA Isolation Kit Tissues (Exiqon, Vedbaek, Denmark) according to the manufacturer's instructions. The quantity and purity of RNA were analyzed spectrophotometrically, and 100 ng of RNA was used for cDNA synthesis, which was synthesized using the Universal cDNA Synthesis Kit II (Exiqon). Spike-in UniSp6 was added to each sample, which allowed to monitor the quality of reaction with reverse transcriptase. Three negative controls of this reaction were prepared using the following reaction mixtures—without reverse transcriptase (no enzyme sample), without RNA template (no template), and using MS2 bacteriophage RNA (Roche, Cat. No. 10165948001) as the template (Mock).

One microgram of total RNA isolated from tissue samples, as mentioned above, was reverse transcribed using the Transcriptor Universal cDNA Master (Roche Diagnostics, Mannheim, Germany) and subsequently used for the evaluation of the expression of *CDH1*. Negative control samples were prepared with the omission of an enzyme and RNA template.

### 2.3. MicroRNA Expression Analysis

Each cDNA sample was diluted with nuclease-free water and mixed with SYBR Green Master Mix with LNA™ primers for the defined miRNAs (Exiqon) according to manufacturer's instructions.

In all tissue samples, the expression of UniSp6 was assessed as a marker of efficiency of cDNA synthesis. Each sample positively verified was qualified for further analysis. The relative quantification (RQ) of five selected miRNAs: *miR-21-3p*, *miR-31-3p*, *miR-125b-1-3p*, *miR-146a-3p*, and *miR-155-5p*, was determined in triplicate using Light Cycler 480® II Instrument and Light Cycler 480® Software 1.5 (Roche Diagnostics). From the potential two reference miRNAs: *miR-103a-3p* and *miR-423-3p*, the latter was chosen as a reference gene due to a constant level of expression without significant differences between analyzed groups (*p* > 0.05).

### 2.4. *CDH1* Expression Analysis

The amplification of each cDNA sample from tissue was performed using LightCycler®480 SYBR Green I Master and sets of primers for *CDH1* (forward: CAGGCTCAAGCTATCCTTGC, reverse: AGTCATGCGTAGTGGTGCAT) according to the manufacturer's protocol. *GAPDH* was used as the reference gene for data normalization (primer forward: CTCTGCTCCTCTGTTCGAC, reverse: GCCCAATACGACCAAATCC).

Relative quantification (RQ) was evaluated in triplicate using Light Cycler 480® II Instrument and Light Cycler 480® Software 1.5 (Roche Diagnostics).

### 2.5. Statistical Analysis

The normality of distribution of miRNA expression in the studied biological samples was assessed using histograms and the Kołmogorov-Smirnov and Shapiro-Wilk tests. Since the distribution was not normal, the intergroup differences in miRNA expression were analyzed using nonparametric tests (Kruskal-Wallis ANOVA by ranks, Mann–Whitney *U* and Wilcoxon rank-sum tests). Correlations between variables were analyzed using Spearman's test. The differences in expression were considered significant at *p* < 0.05. Statistical calculations were performed using Statistica, version 9.0 software (StatSoft, Inc., Tulsa, OK 74104, USA).

## 3. Results

### 3.1. Comparative Analysis of miRNA Expression Profiles in CD, UC, and Healthy Individuals

**Figure 1:**
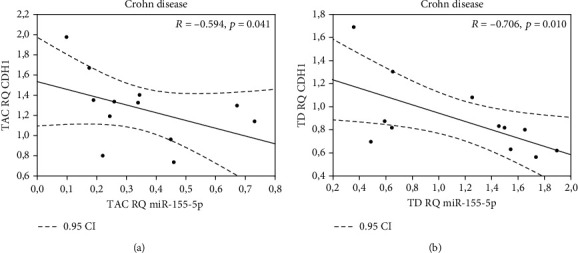
Correlation between (a) *miR-155-5p* and *CDH1* in normal adjacent tissue (TAC); (b) correlation between *miR-155-5p* and *CDH1* in inflamed tissue (TD) of patients with CD.

**Figure 2:**
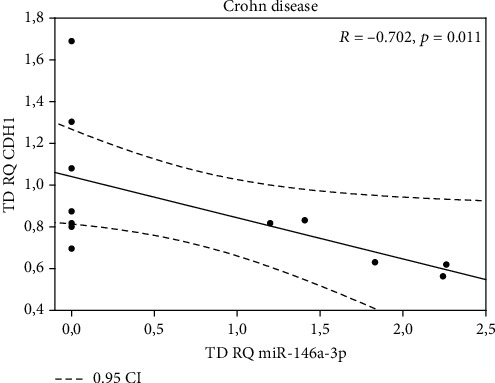
Correlation between *miR-146a-3p* and *CDH1* in inflamed mucosa (TD) of patients with CD.

**Figure 3:**
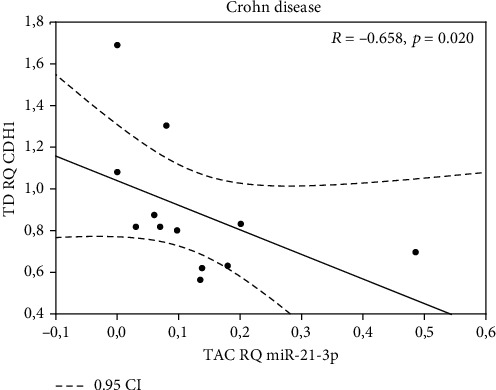
Correlation between *miR-21-3p* in adjacent tissue (TAC) and *CDH1* in inflamed mucosa of patients with CD (TD).

All miRNAs were expressed in examined tissue samples. In biopsy specimens obtained from patients with CD, *miR-21-3p*, *miR-31-3p*, *miR-146a-3p*, and *miR-155-5p* were overexpressed (except *miR-125b-1-3p*) in TD tissues in comparison to TAC samples (Table 2). Our findings indicate that expression of *miR-21-3p*, *miR-31-3p*, *miR-125b-1-3p*, *miR-146a-3p*, and *miR155-5p* was significantly higher in biopsy specimens from inflammation-altered tissue in comparison to the adjacent tissue of patients with UC (Table 3). When analyzing expression of selected miRNA in inflammation-altered tissue of CD or UC patients with biopsy specimens of healthy patients, statistically significant differences were found (Table 4), whereas miRNA analysis did not reveal statistically significant differences in analyzed tissues between the CD and UC patient groups (both, between inflamed mucosa, and adjacent tissues, data not shown). Given the fact that particular miRNAs maintain the intestinal epithelium intact through inhibiting EMT (epithelial to mesenchymal transition) and promoting proliferation of intestinal cells [20, 21] we aimed to test if the expression of the analyzed miRNAs could correlate with the expression of E-cadherin coding gene (*CDH1*), which is a hallmark of EMT [22]. We noted negative correlations between *miR-155-5p* and *CDH1* in both tissues—adjacent normal tissue (*p* = 0.041) and inflamed mucosa (*p* = 0.10) from patients with CD (Figures 1(a) and 1(b)). Similarly, negative correlation was also found between *miR-146a-3p* and *CDH1* in inflamed mucosa only (*p* = 0.011) in the same patient's group (Figure 2). A negative correlation was noted between *miR-21-3p* (*p* = 0.020) in adjacent normal tissue and expression of *CDH1* in the inflamed mucosa of patients with CD (*p* = 0.020) (Figure 3).

**Figure 4:**
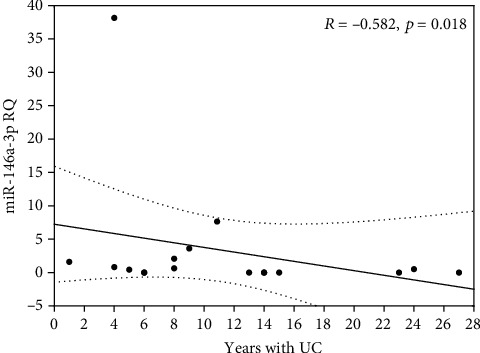
Correlation between *miR-146a-3p* expression (RQ) in inflamed tissue and years with disease in patients with UC (dotted line -0.95 confidence interval).

Taken together, in inflamed mucosa from CD patients, three miRNAs (*miR-21-3p*, *miR-146a3p*, and *miR-155-5p*) were highly expressed, whereas *CDH1* expression was downregulated. Such correlations were not detected in UC patients. In these patients' group (UC), we only found that the duration of disease was significantly and negatively correlated with the expression level of *miR146a-3p* (Spearman's correlation test, *p* = 0.0181, *R* = −0.582, Figure 4). We did not reveal any other correlations between the miRNA expression and patient's clinical status.

## 4. Discussion

Despite years of research, the exact cause of nonspecific inflammatory bowel diseases (IBD) has not been fully explained, and the diagnosis and differentiation between CD and UC remains often difficult. Understanding the molecular mechanisms that modulate the expression of inflammation-related genes is crucial for the detection of new biomarkers of IBD. miRNAs have a huge potential as biological markers, because there are key molecular players in regulating a multitude of cellular processes, including IBD development [23, 24]. Specific miRNA signatures have been observed in IBD, associated with canonical signaling pathways regulating autophagy, inflammation, fibrosis, or EMT [25]. One of the analyzed miRNAs in the present paper is *miR-155* which was shown to inhibit the suppressor of cytokine signaling (*SOCS1*)—a negative regulator of the Janus kinase/signal transducers and activators of transcription (JAK/STAT signaling); thus, elevating the expression of *miR-155* could enhance inflammation in the intestine of IBD patients by immune system perturbation [25, 26]. In our study, we noticed an increased expression of *miR-155-5p* in both inflamed tissue and, most important, also adjacent tissue of patients with CD, suggesting that elevated *miR-155-5p* expression contributes to CD development. We further show that tissues near to CD-related lesions displayed increased *miR-21-3p* expression, and given the fact that involvement of elevated expression of *miR-21* resulted in loss of tight junction proteins and increased barrier permeability [27], the role of these miRNA in pathogenesis of IBD tissues seems to be evident [28]. The molecular background of IBD development was linked to the EMT process with specific miRNA signatures affecting signaling, transcription factors, and mesenchymal or epithelial marker expression [25]. We determined the expression of one of these markers—E-cadherin (*CDH1* gene)—in relation to the miRNA expression pattern in tissues from CD and UC patients.

In our study, we confirmed the lower expression of *CDH1* only in patients with CD, but not in patients with UC, which suggest the involvement of this protein in CD pathogenesis. We noticed that downregulated *CDH1* was negatively correlated with *miR-155-5p* expression in both the inflamed mucosa and adjacent mucosa of such patients, which can assume that this expression pattern may differentiate CD from UC. *miR-155* is primarily expressed in the thymus and spleen and minimally detected in normal physiologic conditions, but its overexpression has been observed in IBD and colorectal cancer (CRC) [29].

Based on our results and the fact that if *miR-155* expression is induced by inflammatory cytokines and toll-like receptor ligands that leads to dysregulations in tissue development and immune response, we can conclude the elevated level of *miR-155-5p* in the adjacent mucosa of patients with CD can reflect the initiation of inflammatory process in the tissue microenvironment. Other overexpressed miRNAs, which were negatively correlated with the expression of *CDH1*, were *miR-146a-3p* and *miR-21-3p*, found in inflamed tissue specimens in patients with CD in our studies. Until now, the influence of selected miRNAs (*miR-9*, *miR-25*, *miR-92a*) for E-cadherin expression has been linked to cancer cell migration and invasion [30] or development of metastasis only [31, 32]. Thereby, our results point out a potential role of the analyzed miRNAs—*miR-21-3p*, *miR-146a-3p*, and *miR-155-5p*—in EMT-mediated CD development or progression. However, further investigation and functional studies are required to confirm this hypothesis.

From the clinical point of view, we noticed that the expression of *miR-146a-3p* was negatively correlated with the duration of disease in UC patients, suggesting that *miR-146a-3p* could be used for monitoring the disease progress, rather than the biomarker which differentiates UC from CD. *miR-146a* is a nitric oxide-responsive miRNA, which amplifies inflammatory responses, including the NF*κ*B pathway [33]. On the other hand, it was reported that the NF*κ*B transcription factor after LPS-stimulation upregulates *miR-125b-1* expression in cultured human epithelial cells [34]. In our studies, we compared expression of *miR-125b-1-3p* in adjacent tissue and inflamed tissue in CD and UC, and we noticed significantly higher expression of this miRNA in UC, but not in CD. Our results are in agreement with the findings of Valmiki et al. [16, 35] who discovered the significantly higher relative expression of *miR-125b* in UC biopsies compared to non-IBD controls. The authors showed that *miR-125b* targets and negatively regulates *TRAF6* and *A20*, the two molecules of NF*κ*B signaling pathway that modulate several genes involved in inflammation. It was suggested that suppression of *TRAF6* and *A20* genes mediated by *miR-125b* could be one of the factors contributing to the chronic inflammation in UC [35].

Our study has some limitations. Firstly, the study is based on bioinformatic prediction of potential interactions between selected miRNA and expression of *E-cadherin*; thus, results have to be confirmed by functional tests. Secondly, the number of patients is limited due to several inclusion and exclusion criteria; thereby, our results need to be validated on a larger population. However, even on analyzing the small cohort of patients, we emphasized the confirmation of correlations between miRNA and *CDH1* expression, which prompt us to continue this study in the future.

To sum up, paying special attention to the relationship between miRNAs and E-cadherin as a core component of colonic epithelium and signaling epicenter that regulates cell behavior [18] will enable developing a new therapeutic strategy to stop or slow down the progression of IBD.

## 5. Conclusions

Our study clearly confirmed that *miR-155-5p*, *miR-146a-3p*, *miR-125b-1-3p*, and *miR-21-3p* are associated with IBD, and their correlations with *CDH1* expression deserve special attention suggesting the involvement of epithelial to mesenchymal transition (EMT) in CD development, but not in UC.

## Figures and Tables

**Table 1:** Characteristic of patients with Crohn disease (CD), ulcerative colitis (UC), and healthy controls.

Characteristic	CD	UC	Control group
No. of patients, *n*	12	16	11
Patients specimens			
Pinch biopsies	24	32	11
Inflammation-altered tissue	12	16	—
Adjacent tissue	12	16	—
Age (y)			
Mean	45,3	49,2	59,4
Min/max	(23/67)	(23/27)	(22/88)
Sex			
Male	6	10	5
Female	6	6	6
Years with disease			
Mean	11,7	11,6	—
Min/max	(1/26)	(1/27)	—
Extension of disease, *n*			
Left-sided colitis	—	8	—
Proctitis	—	3	—
Pancolitis	—	5	—
Site of disease, *n*			
Ileal	3	—	—
Colitis	5	—	—
Ileocolitis	4	—	—
CDAI activity, *n*			
Mild	5	—	—
Moderate	5	—	—
Severe	2	—	—
Mayo score, *n*			
1	—	4	—
2	—	6	—
3	—	6	—
Smoking, *n*			
Yes	9	4	3
No	3	12	8

**Table 2:** miRNA expression (RQ) in inflamed tissue and adjacent tissue of patients with CD.

Crohn disease (CD, *n* = 12)					
RQ	Inflamed tissue		Adjacent tissue		*p* ^∗^
	Mean ± SD	Median (min-max)	Mean ± SD	Median (min-max)	
*miR-155-5p*	0.348 ± 0.197	0.299 (0.093-0.731)	1.147 ± 0.557	1.354 (0.356-1.894)	**0,006040**
*miR-146a-3p*	0.112 ± 0.388	0 (0-1.345)	0.745 ± 0.965	0 (0-2.262)	**0,043115**
*miR-125b-1-3p*	0.677 ± 0.965	0 (0-8.123)	1.805 ± 1.591	1.277 (0-4.872)	0,050461
*miR-31-3p*	0.843 ± 0.177	0 (0-6.835)	7.142 ± 9.817	6.054 ± (0 − 36.38)	**0,009926**
*miR-21-3p*	0.123 ± 0.131	0.089 (0-0.486)	2.335 ± 0.131	2.282 ± (0.325 − 4.181)	**0,002218**

Bold *p* values are statistically significant. ^∗^Wilcoxon test; SD: the standard deviation of the mean.

**Table 3:** miRNA expression (RQ) in inflamed tissue and adjacent tissue of patients with UC.

Ulcerative colitis (UC, *n* = 16)					
RQ	Inflamed tissue		Adjacent tissue		*p* ^∗^
	Mean ± SD	Median (min-max)	Mean ± SD	Median (min-max)	
*miR-155-5p*	0.282 ± 0.117	0.258 (0.061-0.504)	1.455 ± 0.604	1.460 (0.493-2.514)	**0,000438**
*miR-146a-3p*	0.407 ± 1.126	0 (0-3.739)	3.530 ± 9.491	0.482 (0-38.150)	**0,028403**
*miR-125b-1-3p*	0.276 ± 0.651	0 (0-2.064)	2.0128 ± 1.468	1.553 (0-5.497)	**0,000982**
*miR-31-3p*	0.154 ± 0.369	0 (0-1.249)	3.310 ± 6.096	0.909 (0-19.230)	**0,007133**
*miR-21-3p*	0.126 ± 0.158	0.082 (0-0.462)	2.703 ± 1.417	2.158 (1.070-5.669)	**0,000438**

Bold *p* values are statistically significant. ^∗^Wilcoxon test; SD: the standard deviation of the mean.

**Table 4:** miRNA expression (RQ) in tissues (TD) of patients with Crohn disease and the control group (healthy individuals).

TD RQ	Crohn disease (*n* = 12) vs. control group (*n* = 11)	Ulcerative colitis (*n* = 16) vs. control group (*n* = 11)
	*p*	*p*
*miR-155-5p*	**0,000506**	**0,000087**
*miR-146a-3p*	0,096567	**0,015608**
*miR-125b-1-3p*	**0,004639**	**0,001459**
*miR-31-3p*	**0,000282**	**0,000946**
*miR-21-3p*	**0,000093**	**0,000016**

Mann–Whitney *U* test; bold *p* values are statistically significant.

## Data Availability

The data that support the findings of this study are available from the corresponding author [MG], upon reasonable request.
